# Genetic variation in olfactory pathways associated with host-seeking behavior in natural populations of *Anopheles minimus*, a primary malaria vector in western Thailand

**DOI:** 10.1186/s13071-025-07029-x

**Published:** 2025-09-24

**Authors:** Kanchon Pusawang, Daibin Zhong, Patchara Sriwichai, Yudthana Samung, Atiporn Saeung, Kittipat Aupalee, Pradya Somboon, Anuluck Junkum, Somsakul Pop Wongpalee, Jassada Saingamsook, Jetsumon Sattabongkot, Liwang Cui, Guiyun Yan

**Affiliations:** 1 Parasitology and Entomology Research Cluster (PERC), Faculty of Medicine, Chiang Mai University, Chiang Mai, 50200 Thailand; 2 Department of Population Health and Disease Prevention, University of California, Irvine, CA 92697 USA; 3 Department of Medical Entomology, Faculty of Tropical Medicine, Mahidol University, Bangkok, 10400 Thailand; 4 Department of Microbiology, Faculty of Medicine, Chiang Mai University, Chiang Mai, 50200 Thailand; 5 Mahidol Vivax Research Unit, Faculty of Tropical Medicine, Mahidol University, Bangkok, 10400 Thailand; 6 Department of Internal Medicine, Morsani College of Medicine, University of South Florida, Tampa, FL 33612 USA

**Keywords:** Mosquito, Whole-genome sequencing, Single-nucleotide polymorphism, Olfaction

## Abstract

**Background:**

Malaria transmission hinges on infected *Anopheles* mosquitoes biting humans, with carbon dioxide (CO_2_), host odor, and body heat acting as key attractants. Along the Thai–Myanmar border, *Anopheles minimus* (the Funestus Group), a primary malaria vector, exhibits a stronger preference for human hosts than species of the Maculatus Group. Elucidating the genetic basis of this feeding behavior is essential for improving malaria control strategies.

**Methods:**

Wild *Anopheles* mosquitoes were collected in Tha Song Yang district, Tak province, Thailand, from July 2019 to November 2020, using cow-baited traps, human landing catches, and Center for Disease Control (CDC) light traps. Specimens were identified morphologically and confirmed by Sanger sequencing of the cytochrome* c* oxidase subunit 1 (*cox1*) gene. We then performed whole-genome sequencing on *An*. *minimus* females categorized by host-seeking behavior: cow-baited collection (COW), human landing indoor (HLI), and human landing outdoor (HLO) to investigate the genetic determinants of host preference.

**Results:**

*Anopheles minimus* females accounted for 25% of total samples (504/1,997). *Cox1* sequencing revealed 143 unique haplotypes among 287 specimens, forming two major phylogenetic lineages, A (181 sequences) and B (106 sequences), suggestive of potential cryptic diversity. Whole-genome sequencing of *An*. *minimus* Lineage A from COW, HLI, and HLO groups yielded 12,659,785 variants. After filtering, 68,975 non-synonymous single-nucleotide polymorphisms (nsSNPs) remained. Comparing allele frequencies across the three pooled groups (FDR-adjusted *p*-value < 0.001) yielded 2,629, 2,948, and 4,369 significant nsSNPs, respectively. Gene Ontology (GO) analysis of genes harboring these nsSNPs showed strong enrichment for olfaction-related terms. The top six nsSNPs with olfactory annotations from each group comparison were selected for validation; Sanger sequencing confirmed their association with host-seeking preference. The VectorBase gene IDs for these candidate nsSNPs are AMIN001807, AMIN001339, AMIN003886, AMIN000912, AMIN003926, AMIN011060, AMIN002342, and AMIN015480.

**Conclusions:**

The observed significant genomic variance in field-collected *An*. *minimus* females, categorized by collection methods (reflecting host-seeking behavior), proposes a genetic underpinning for these behavioral variations. Differential nsSNPs within olfactory pathway genes might be functionally linked to host-seeking in this important malaria vector.

**Graphical Abstract:**

**Figure:**
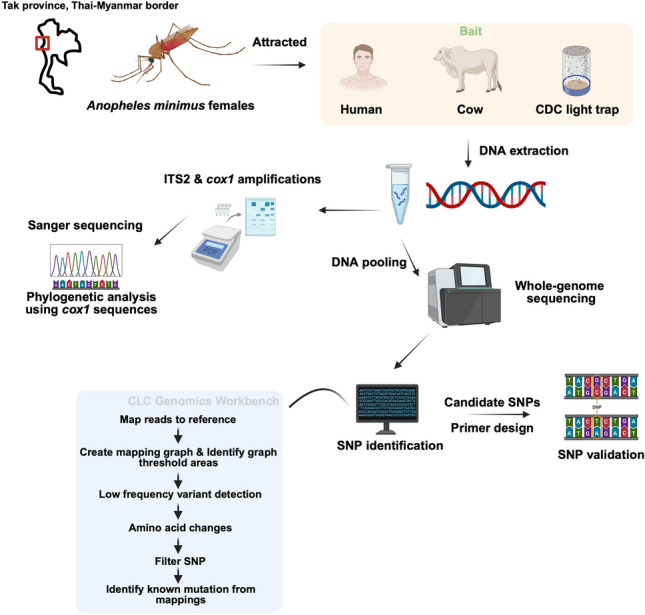

**Supplementary Information:**

The online version contains supplementary material available at 10.1186/s13071-025-07029-x.

## Background

Malaria, caused by *Plasmodium* spp., Generally results in 600,000 deaths every year [1]. Certain female *Anopheles* mosquitoes transmit such pathogen to humans by biting [2]. The need of essential nutrients for egg development obligates them to feed on blood from hosts [3]. Thus, their innate biting behavior play a significant role in the disease transmission. To locate the hosts, they rely heavily on their robust olfactory organization [4, 5]. Carbon dioxide (CO_2_), host odor, and body heat are utilized as main cues, integrated with other sensory signals derived from humidity, mechanical effect, and visual inputs [6, 7]. Mosquitoes and other insects perceive those cues using peripheral appendages covered with sensilla [8]. Several forms of sensilla have been established on antennae, maxillary palps, proboscis, and tarsi of mosquito vectors [9–17]. Moreover, they are innervated by neurons that express olfactory receptors [18–22]. Zeroing in on insect olfactory receptors, these ligand-gated ion channels are encoded by three main gene classes: odorant receptors (ORs), ionotropic receptors (IRs), and gustatory receptors (GRs). Conventional ORs form a heterotetrameric complex with the highly conserved co-receptor Orco [23–27]. Ionotropic receptors are a variant subfamily of ionotropic glutamate receptors comprising IR tuning receptors and one of the three identified co-receptors, Ir8a, Ir25a, and Ir76b [28, 29]. A spectrum of odorants elicits responses from ORs and IRs [30, 31]. Strikingly, some IRs also detect temperature and humidity shifts [32–35]. In contrast to the previously mentioned receptors, the GR coreceptors have not yet been observed. The coreceptor-independent GRs are highly sensitive to CO_2_ by creating a three-GR complex [36–39]. Information via olfactory sensory neurons is encoded into an odor map inside the glomeruli within the antennal lobe and the higher brain centers, respectively [4].

Apart from well-studied anthropophilic vectors such as the dengue fever mosquito, *Aedes aegypti* [40] and the African malaria mosquito, *Anopheles gambiae* [41], most mosquito species tend to prefer animals as blood sources [42]. The increased expression of olfactory receptors assists mosquitoes in distinguishing between human and non-human hosts more precisely [42–46]. Hence, disruption of this fascinating system has received more attention over the past decade. Mutating the functional receptors to modulate host preference has been implemented by interrupting the expression of *Orco* [47], IR co-receptor genes (*Ir8a*, *Ir25a*, or *Ir76b*) [29, 48], and *Gr3* [49]. The results from these works show a moderate loss of attraction to humans, but mutant mosquitoes still retain their anthropophilia. In field settings, environmental conditions profoundly affect mosquito feeding behavior, from generalists, who feed on a wide range of vertebrates, to host-specific mosquitoes or specialists [50–52]. Such adaptive feeding behavior could overcome the inherent traits in unfavorable situations, including the availability of preferred hosts [53, 54], the coverage of insecticide-treated interventions [55], and the parasite manipulation [56, 57].

Little is known about the underlying mechanisms in host-seeking behavior in field-collected mosquitoes, which could possibly be the game changer for new vector control strategies. In this study, we conducted a comparative analysis of *An*. *minimus* genomes from natural populations with different types of host preferences based on collection methods. Female *An*. *minimus* was chosen due to its strong anthropophilic behavior and vector competence compared with other vector species at the Thai–Myanmar border [58]. We performed a phylogenetic analysis, on the basis of *cox1*, and detected a new lineage of *An*. *minimus*, suggesting a possible unknown species. To assess the presence of the single-nucleotide polymorphisms (SNPs) within the olfactory-related genes, whole-genome sequencing was performed to generate genomic data.

## Methods

### Study site

The mosquitoes were collected within three hilly villages in Tha Song Yang district, Tak province: Suan Oi (SO, 17° 32′ 26.484'' N, 97° 56′ 16.908'' E), Pha Man (PM, 17° 32′ 22.596'' N, 97° 56′ 22.416'' E), and Komonae (KN, 17° 32′ 4.236'' N, 97° 57′ 9.684'' E) at the Thai–Myanmar border. The temperature typically ranges between 16 °C and 36 °C, and the average annual precipitation is 74.2 mm [59]. Malaria is endemic in these neighborhoods. Most of the indigenous cases are dominated by *Plasmodium vivax* while *P*. *falciparum* infections are rare and typically defined as imported cases [60]. The principal malaria vectors in this region are *An*. *minimus*, *An*. *maculatus*, and *An*. *dirus* [61–63].

### Mosquito collection

To investigate the phenotypic host preference of *Anopheles* mosquitoes in malaria-endemic areas, collections of mosquitoes were carried out by three methods, cow bait, human bait, and Center for Disease Control (CDC) light traps. We tried to mimic the natural conditions by assigning the collection method in the study sites to represent the host seeking behavior of collected mosquitoes. Considering this is more convenient strategy to carry out the behavioral study in the field trials, there are several external factors (e.g., seasonal dynamics and weather conditions) that could affect the host preference and should not be overlooked. Notably, the availability of the preferred hosts is one of the significant factors that consistently concerns us in field studies. To minimize these confounding effects, the collections were conducted simultaneously, and all baits were placed exclusively outside the village boundaries at standardized distances.

Collections were conducted four times a month (on 5 consecutive nights) between 18.00 and 06.00 h from June 2019 to January 2020, and October to December 2020. These periods showed the highest abundance of *An*. *minimus* [61]. Collections were carried out by well-trained villagers as paid-volunteers. All participants were given a full explanation of the test procedure both verbally and in written form prior to human landing catch. With the aim of obtaining anthropophilic mosquitoes from the field settings, each volunteer was assigned as human bait inside (HLI) or outside the house (HLO). The distance between HLI and HLO was 20 m apart. They sat on the floor and exposed their legs. Each collection time was 45 min with a 15-min break. Mosquitoes that landed on the skin were caught with an aspirator, and placed in net-covered cups, provided with sugar-soaked cotton. The cups were labeled by date, location, and hour of collection. For cow-baited collection (COW), a cow was tethered overnight inside a net (3.6 × 3.5 × 2 m) with a zippered opening, 300 and 400 m from the villages. Blood-engorged females resting inside the net were collected by an aspirator and transferred to mosquito cups in the following morning. These samples were classified as zoophilic. In addition, CDC Light traps and 6-V battery with dry ice (*n* = 28, BioQuip model 2836BQ, USA) were utilized to collect mosquitoes from selected houses (*n* = 14). The traps were hung approximately 1.5 m above the ground, either indoors (LTI) or 10–20 m away from the houses (LTO). Mosquitoes in the traps were transferred to mosquito cups each morning. The mosquito cups were delivered to the laboratory in the field station for morphological identification using a taxonomic key [64].

### DNA extraction and molecular identification

Genomic DNA was extracted from the whole body of each *An*. *minimus* female using the PureLink^™^ Genomic DNA Mini Kit (Invitrogen, Carlsbad, CA), according to the manufacturer’s instructions. DNA quantity was evaluated using the Nanodrop 2000 (Thermo Fisher Scientific, Waltham, MA). To distinguish *An*. *minimus* *s.s.* from other sibling species in the Minimus Complex, the ITS2 region was amplified as previously described [65]. The partial *cox1* gene was used for DNA barcoding and haplotype mapping. The specific primers and protocol were described previously with minor modifications [66]. The amplified products were electrophoresed on 1.5% agarose gel and stained using Ultrapower^™^ dye (BioTeke, Bejing, China). Positive *cox1* PCR products were sent for Sanger sequencing at First BASE Laboratories (Selangor, Malaysia).

### Phylogenetic analyses

The sequences of *cox1* gene were then analyzed to determine the intra- and interspecific variation within the *An*. *minimus* populations. This mitochondrial gene is posited as a standard gene for DNA barcoding in invertebrates, and its resolution prevails over other nuclear genes [67]. The raw sequences were assembled and aligned using Geneious Prime version 2021.0.3 and compared with *cox1* references using BLAST in NCBI. Haplotype diversity was determined by DnaSP6 [68]. The mosquito reference sequences including *An*. *minimus* (GenBank accession HQ877373) and *An*. *harrisoni* (HQ877375) were aligned with consensus sequences to construct a phylogenetic tree in MEGA version 7.0 with MUSCLE under default parameters [69]. The Kimura 2-parameter (K2P) model was applied for estimating genetic distances [70]. The jModelTest version 2.1.10 was used to assess the best-fit evolutionary model, which was HKY + I + G, for phylogenetic inference based on the maximum likelihood (ML) method with 1,000 bootstrap replicates [71].

### Library preparation and whole-genome sequencing

Intrinsic factors including the variation of olfactory genes could be noticeable in wild mosquitoes, which perpetually confront dynamic environment. To begin to justify this assumption, we carried out WGS using pooled *An*. *minimus* samples from the lineage A on the basis of the phylogenetic analysis. Given Pool-seq (whole-genome sequencing of pools of individuals) is more unbiased and cost-effective than sequencing individuals separately for population genomic analyses, the influence of the pool size and the limitations are discussed [72, 73]. The equal amount of DNA from each sample should be optimized before pooling to thoroughly represent individuals in the pool [74]. Very small pools show significant bias in allele frequency estimates [72]. Its accuracy increases with a large pool size and high sequencing coverage [75]. In this study, we pooled 10 individuals for WGS, which is empirically sufficient to assess colony-level phenotypes in *Cataglyphis niger* ants [76]. Since natural *Anopheles* mosquitoes exhibit high behavioral plasticity, we pooled samples to reduce allele frequency bias compared with individual sequencing and to derive a consensus for each group. However, larger pool sizes and greater sequencing depth are required to minimize inherent allele frequency estimation errors in pooled sequencing.

DNA quality and quantity of each individual mosquito were assessed using Nanodrop 2000 (Thermo Fisher Scientific, Waltham, MA) and the Qubit^™^ fluorometer (Invitrogen, Carlsbad, CA). DNA integrity was checked by agarose gel electrophoresis. Samples that passed quality control from the COW, HLI, and HLO groups were pooled by mixing equal amounts of DNA (~ 100 ng per sample) to create three pools of 10 samples each. The nine pooled DNA samples were adjusted to a final DNA amount of ~ 1 µg and sent to Admera Health Biopharma Services (New Jersey, USA) for library preparation and whole-genome sequencing. Briefly, libraries were constructed using the KAPA Hyper Prep kit without PCR (Roche, Basel, Switzerland). The quality and quantity of the libraries were determined on the Agilent TapeStation (Agilent Technologies, Santa Clara, CA) and in the QuantStudio System (Applied Biosystems, Foster City, CA). The Libraries were sequenced on the Illumina NovaSeq 6000 sequencer (Illumina, San Diego, CA) and 150-bp paired-end reads were generated.

### Sequence read alignment and SNP identification

FASTQ reads were trimmed and adapters removed by Trimmomatic version 0.32 to a Phred score of 30 [77]. Then, the reads were filtered on the basis of their quality using the Illumina pipeline function to trim those of low-quality reads and filter out failed ones in the CLC Genomics Workbench version 12.0.3 software (CLCbio, Aahus, Denmark). Cleaned reads of each pooled sample were mapped to the *An*. *minimus* reference genome, AminM1.9, GCA_000349025.1 (http://www.vectorbase.org) under default parameters in the CLC Genomics Workbench. Variant calling was carried out on a mapped read using the Low Frequency Variant Detection tool in the CLC Genomics Workbench. It applied modified parameters as follows: required significance = 1%, ignore positions with coverage above = 2,700,000, ignore broken pairs = yes, minimum coverage = 540 (based on 90 samples with at least 6 reads for each sample), minimum count = 54, minimum frequency = 10%, base quality filter = no, read direction filter = no, relative read direction filter = yes, read position filter = no, remove pyro-error variants = no. Single nucleotide polymorphisms (SNPs) with coverage < 10,000 were chosen. Non-synonymous variants were extracted using the Amino Acid Changes tool in the CLC Genomics Workbench and considered as a known variant template. The allele frequency of each pool was generated using the Identify Known Mutations from mappings tool in the CLC Genomics Workbench, and the variant file as described above was employed. The parameters were adjusted as follows: minimum coverage = 10, detection frequency = 20%, ignore broken pairs = no, ignore nonspecific matches = no. The excel files of resulting data were exported. The read counts and coverages from each pooled sample collected by the same method were combined in Microsoft Excel. SNPs with coverage > 1,000 were filtered out and then imported into *R* for Fisher’s exact test [78]. Differential SNPs were annotated with AminM1_mRNA and AminM1_gene_ontology using merge() and left_join(), respectively, in the R base version 4.4.2 [78]. Gene ontology (GO) analysis was conducted using the web-based tool ShinyGO version 0.82 (http://bioinformatics.sdstate.edu/go/) [79].

### SNP validation and multiple correspondence analyses (MCA)

Significantly differential SNPs that existed in both COW versus HLI and COW versus HLO were identified using inner_join() in the R base version 4.4.2 [78]. These variants were sorted in ascending order on the basis of the FDR-adjusted *p*-values. The top six SNPs associated with olfactory function were selected for genotyping. To validate the allele frequency of the chosen SNPs within individual samples, specific primers were designed by Primer3 version 4.1.0 [80]. Target regions were extracted from the reference Genome using SeqKit version 2.5.1 [81]. Each PCR was carried out in a total volume of 20 µL containing 0.4 U of Platinum™ *Taq* DNA polymerase (Invitrogen, Carlsbad, CA), 1X of PCR buffer, 2 mM of MgCl_2_, 0.2 mM of dNTPs, 0.2 µM of each primer, and 1 µL of DNA template. The conditions of amplification consisted of initial denaturation at 94 °C for 2 min, 35 cycles of denaturation at 94 °C for 30 s, annealing at 56 °C for 30 s, and extension at 72 °C for 30 s, with a final extension at 72 °C for 5 min. After performing DNA gel electrophoresis, amplified products were sent to First BASE Laboratories (Selangor, Malaysia) for Sanger sequencing. The FactoMineR version 2.11 [82] and factoextra version 1.0.7 [83] packages were chosen for performing MCA.

### Statistical analyses

Fisher’s exact tests were conducted to examine the differences in variant allele frequency between three groups using fisher.test() function in stats package version 3.6.2 [78]. Statistical significance was determined at *p* < 0.001. The false discovery rate (FDR) adjusted *p*-value was computed by p.adjust (*p*-values, method = “BH”) function using the Benjamini Hochberg method [84].

## Results

### Female *An*. *minimus* exhibits the highest anthropophilic behavior based on human landing catch method

**Fig. 1:**
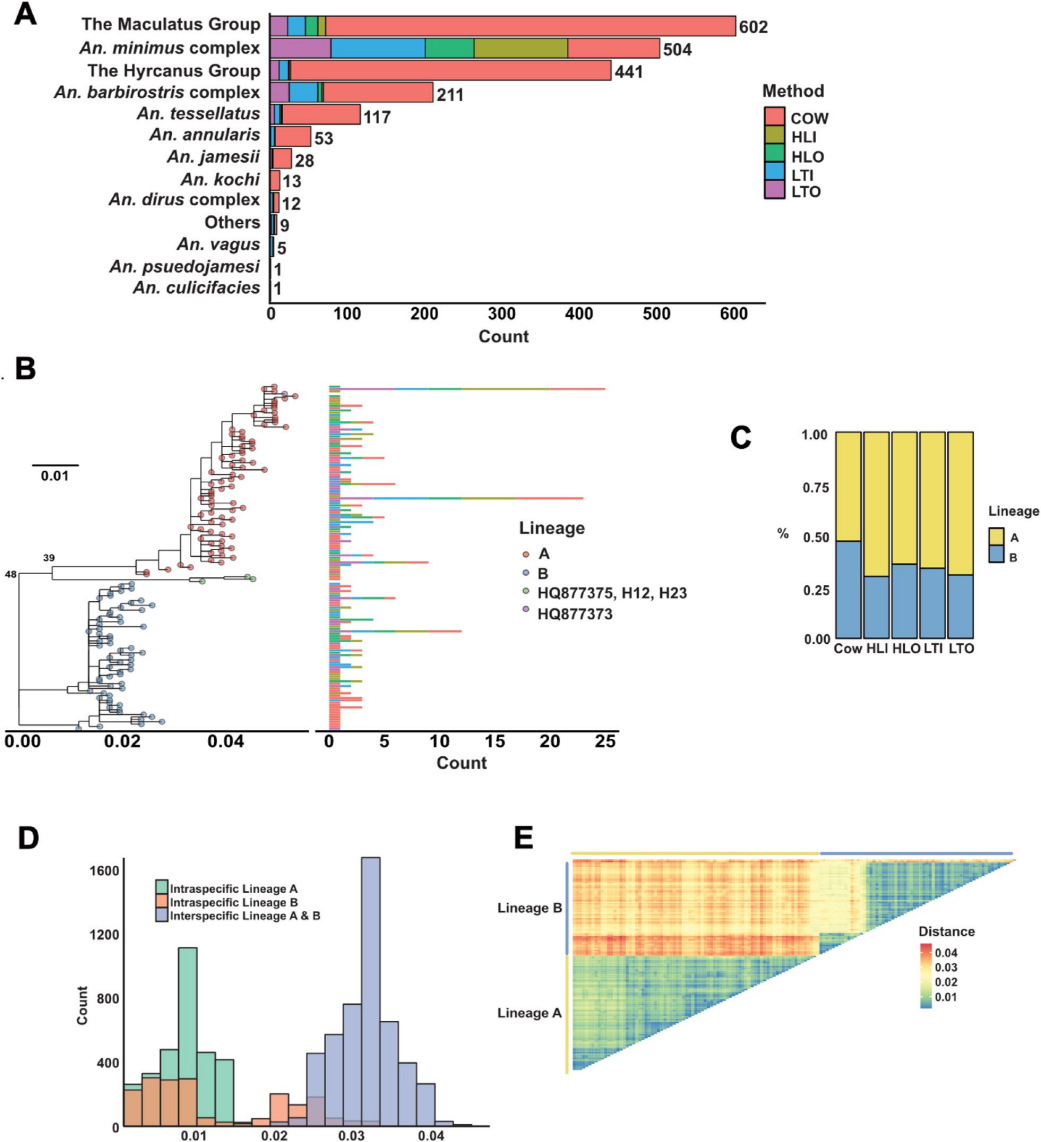
*Anopheles* composition and *cox1* sequence analyses **A** A stacked bar chart displaying species abundance of *Anopheles* female samples with collection methods. **B** Maximum likelihood phylogenetic tree based on *cox1* Gene showing 143 haplotypes and two lineages that are lineage A (red dots) and lineage B (blue dots). HQ877375 (*An*. *harrisoni*), H12 (haplotype No. 12), and H23 (haplotype No. 23) are indicated in green nodes while HQ877373 (*An*. *minimus*) is denoted in purple. The scale bar represents 0.01 substitution of the nucleotide. A side stacked bar chart represents the number of each haplotype with collection methods. **C** The proportion of lineages A and B in each collection method. **D** Histogram showing the intra- and interspecific distances of haplotypes. **E** Heatmap showing the intra- and interspecific distances of haplotypes. *COW*  cow-baited collection, *HLI* human landing indoor, *HLO* human landing outdoor, *LTI*   light trap indoor, *LTO* light trap outdoor.

A total of 1,997 females of *Anopheles* spp. were collected, with the Maculatus Group comprising 30% and the *An. minimus* complex 25% of the catches (Fig. 1A and Table S1). Of 228 human-baited samples, 184 (81%) were morphologically identified as members of the *An*. *minimus* complex. In contrast, only 9% of animal-baited samples belonged to this complex, compared with 38% for the Maculatus Group and 30% for the Hyrcanus Group. There were statistically significant associations between the number of captured *An*. *minimus* complex and collection method (Fisher’s exact test, two-tailed *p* < 0.05), except for indoor versus outdoor CDC light trap comparison. Conversely, the Maculatus Group exhibited a strong preference for animal hosts, with the highest number of cow-collected samples among the methods (Fisher’s exact test, two-tailed *p* < 0.05).

### The plausible two lineages are present in the *An*. *minimus* population

Our initial analyses revealed no significant variation of ITS2 sequences of *An*. *minimus* specimens in the area (unpublished data), but high diversity was observed in the *cox1* sequences. For phylogenetic analysis, 287 randomly selected *An*. *minimus* samples underwent *cox1* sequencing, yielding 143 haplotypes from 616-bp alignment (Table S2). The phylogenetic tree showed three unique clades, consisting of two major ones flanking a minor one (Fig. 1B and Fig. 1C). Most haplotypes fell in lineage A or Lineage B. Lineage A was detected in 181 sequences, whereas Lineage B had a smaller number with 106 sequences. The H12 and H23 haplotypes were closely related with *An*. *harrisoni* (HQ877375) with 0.9 and 1% Genetic distances,  respectively. The mean intraspecific distance of each was less than 1.5% while the mean interspecific value was above 3% (Fig. 1D, E). We also noted that the maximum intraspecific distance within lineage B group (3.9%) exceeded the minimum interspecific value (2%), resulting in the subtle overlapping area between intraspecific zone of lineage B and interspecific group, and also the ambiguity in the *cox1* interpretation.

### Whole-genome analysis reveals significant SNPs in olfactory pathway genes between each group comparison

**Fig. 2:**
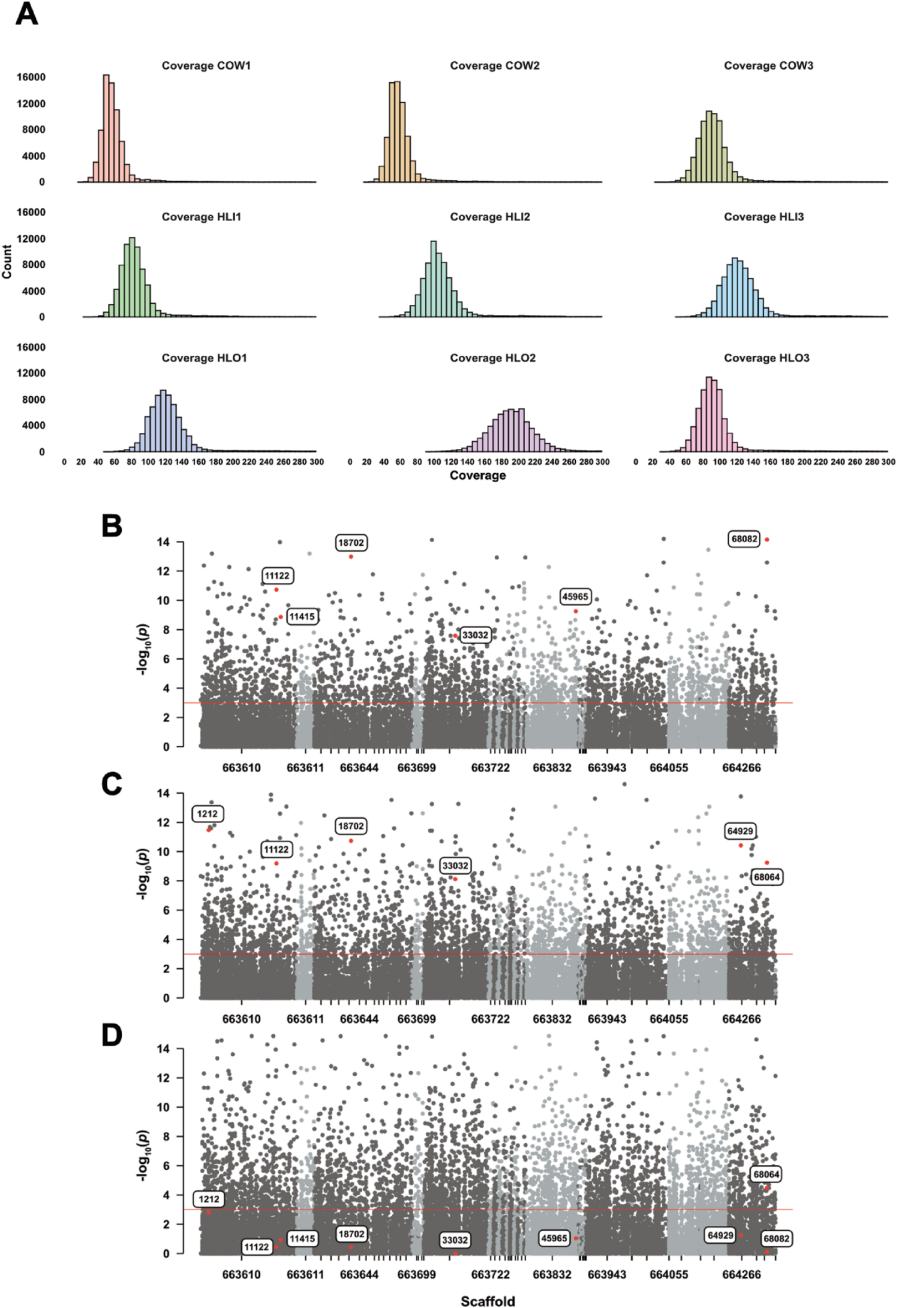
Whole-genome sequencing analyses and SNP discovery **A** The distribution of single nucleotide variant (SNP) coverage in the nine pooled *An*. *minimus* mosquitoes. Three replicates were conducted in each group. **B**–**D** Manhattan plots of differential non-synonymous single-nucleotide polymorphisms in each group comparison: **B** COW versus HLI. **C** COW versus HLO. **D** HLI versus HLO. The candidate SNPs are highlighted in bold. The red horizontal line indicates a significance threshold of *p*-value = 1 × 10^–3^*.* *COW* cow-baited collection, *HLI* human landing indoor, *HLO* human landing outdoor.

Cleaned reads of each pooled sample were mapped to the reference genome (AminM1.9, GCA_000349025.1) simultaneously and 1,813,223,170 paired-end reads were Generated, resulting in an averaged and individual coverage depth of 676.6 × and 7.5 × , respectively. Individual mappings were also performed by mapping individual cleaned reads to the AminM1.9 (Table 1 and Fig. 2A). Normalized combined reads were employed for variant calling, which identified 12,659,785 variants. Of these, 78,635 were non-synonymous and located in the coding regions, eliciting amino acid changes. Since non-synonymous single-nucleotide polymorphisms (nsSNPs) were our target variants, synonymous (sSNP) variants with coverage < 10,000 were filtered out, resulting in 68,975 nsSNPs. SNP rates were filtered using the allele frequency of 0.10 (Table 1). To identify known nsSNPs from each pooled sample, prior ones were used as a variant track to call them from individual mappings, thus creating nine variant files. Read counts and coverages were extracted and combined into three groups (g1 = COW, g2 = HLI, g3 = HLO) on the basis of the collection methods. The nsSNPs with coverage > 1,000 were removed. After computing the *p*-values using Fisher’s exact tests, we discovered 2,629, 2,948, and 4,369 differential nsSNPs from g1 versus g2, g1 versus g3, and g2 versus g3, respectively (Fig. 2B–D and Tables S3–S5).

**Table 1:** Summary of whole-genome sequencing of *An*. *minimus* with different biting behaviors

Group	Replicate	Raw read	Cleaned read	Mapped read	Mean SNP count (95% CI)	Mean SNP coverage (95% CI)	SNP rate (95%CI)	Mapped percentage (%)	Coverage depth (1x)
COW	I	252,112,574	182,377,695	112,620,117	26.48 (26.18–26.78)	63.60 (63.17–64.03)	0.50 (0.49–0.52)	61.80	4.2
	II	262,733,338	194,882,349	117,219,841	27.16 (26.85–27.47)	65.64 (65.17–66.11)	0.50 (0.49–0.51)	60.10	4.4
	III	335,299,620	241,732,431	177,521,836	43.58 (43.09–44.06)	103.55 (102.84–104.27)	0.51 (0.49–0.52)	73.40	6.6
HLI	I	263,387,232	189,878,989	157,731,517	38.92 (38.51–39.32)	92.95 (92.35–93.55)	0.51 (0.49–0.52)	83.10	5.9
	II	354,147,544	244,819,317	202,737,145	49.69 (49.19–50.20)	118.60 (117.84–119.35)	0.51 (0.50–0.53)	82.80	7.6
	III	421,074,322	295,386,826	242,756,494	58.78 (58.16–58.97)	139.87 (138.95–140.79)	0.51 (0.50–0.53)	82.20	9.1
HLO	I	415,798,774	302,739,867	240,335,443	58.34 (57.71–58.97)	138.78 (137.85–139.71)	0.51 (0.50–0.53)	79.40	9
	II	661,669,770	469,656,355	383,986,668	92.51 (91.57–93.45)	220.76 (219.36–222.17)	0.52 (0.51–0.54)	81.80	14.3
	III	305,731,560	218,547,031	178,314,109	42.87 (42.43–43.30)	102.50 (101.83–103.17)	0.51 (0.49–0.52)	81.60	6.7

*COW* cow-baited collection, *HLI* human landing indoor, *HLO* human landing outdoor

**Fig. 3:**
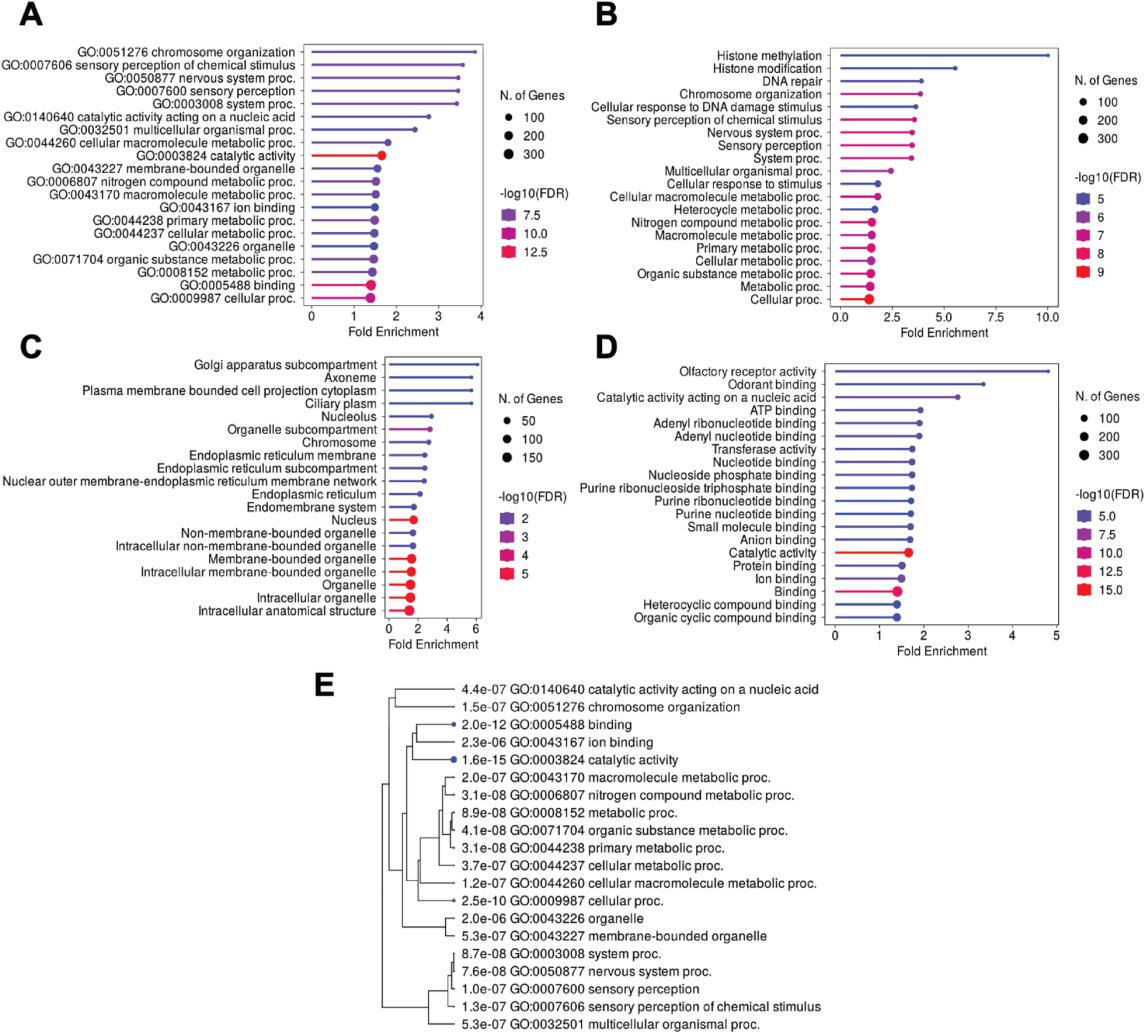
Gene ontology (GO) enrichment analyses of mutual nsSNPs between COW versus HLI and COW versus HLO comparisons (602 unique protein-coding genes). **A**–**D** Lollipop charts showing top 20 pathways of all gene sets (**A**), biological processes (**B**), cellular components (**C**), and molecular functions (**D**), respectively. **E** The hierarchical clustering tree of the correlation of top 20 pathways. The size of the blue nodes indicates the significant level of *p*-value.

Among 798 mutual nsSNPs between g1 versus g2 and g1 versus g3 comparisons, 602 unique protein-coding genes were identified. To investigate the functional attributes and activities of these genes, we categorized the gene characteristics into Gene Ontology (GO) terms and performed GO enrichment analysis with a cutoff of FDR < 0.05 using ShinyGO version 0.82. The top three most enriched GO categories in all given data sets were GO:0051276 chromosome organization, followed by GO:0007606 sensory perception of chemical stimulus and GO:0050877 nervous system process, respectively (Fig. 3A, E and Table S6). While defining GO in three aspects (Biological Process: BP, Cellular Component: CC, and Molecular Function: MF), there were 152 BP, 46 CC, 88 MF enrichments, respectively (Fig. 3B–D and Tables S7–S9). Intriguingly, we noted that GO:0004984 olfactory receptor activity and GO:0005549 odorant binding were the most highly enriched in molecular function category (Fig. 3D). This points to the magnitude of olfactory-related gene families such as olfactory receptor and odorant-binding protein genes in the context of host-seeking behavior driven by the genetic polymorphisms.

The nsSNPs annotated with olfactory receptors (ORs, IRs, and GRs) accounted for 4.3% (113) and 5.0% (148) of the total in g1 versus g2 and g1 versus g3, respectively, whereas g2 versus g3 contained 4.9% (213) of such SNPs. In addition, we found 60 mutual nsSNPs between g1 versus g2 and g1 versus g3. To validate the occurrence of significant variants in each individual sample, mutual nsSNPs were ranked in ascending order according to *p*-values, and the top six nsSNPs were chosen for Sanger sequencing. The following VectorBase gene IDs were used: AMIN001807, AMIN001339, AMIN003886, AMIN000912, AMIN003926, AMIN011060, AMIN002342, and AMIN015480 (Table 2). We noted that in HLI versus HLO comparison, most of selected nsSNPs fell under the threshold except for SNP No. 68064 (Fig. 2D).

**Table 2:** The selected SNPs associated with host-seeking preference in *An*. *minimus* for Sanger sequencing

SNP No.	Scaffold	Region	Gene ID	Amino acid change	Gene description	Ortholog/similar protein
68082	KB664277	3390629	AMIN001807	Tyr104Phe	Or18	–
18702	KB663644	679476	AMIN001339	Thr428Met	Or9	- ACMO_003324 odorant receptor Or1-like (*An.* *coluzzii*) - AGAP008894 Or65 (*An*. *gambiae*)
11122	KB663610	24859250	AMIN003886	Val430Ile	Ir139	- ASTE003155 Ir139 (*An*. *stephensi*) - AFUN004207 Ir139 (*An*. *funestus*)
45965	KB663832	16299463	AMIN000912	Ala264Gly	Ionotropic receptor	- ACUA025671 (*An*. *culicifacies*) - ASTE004650 (*An*. *stephensi*)
11415	KB663610	26266674	AMIN003926	Asp74His	Or410	- AGAP002639 Or39 (*An*. *gambiae*)
33032	KB663721	10410166	AMIN011060	His126Pro	Or33	- ACUA020367 Or33 (*An*. *culicifacies*)
1212	KB663610	2623713	AMIN002342	Asp59Gly	Gr60	- ASTE005723 Gr60 (*An*. *stephensi*)
64929	KB664266	3985239	AMIN015480	Arg119His	Gustatory receptor	- AGAP028572 (*An*. *gambiae*) - ACON028572 (*An. coluzzii*)
68064	KB664277	3389955	AMIN001807	Leu250Phe	Or18	–

Gene description is derived from VEuPathDB. Ortholog and similar protein section is based on protein similarity (> 50%) in the well-known anopheline members from OrthoMCL and UniRef databases.

### Multiple correspondence analysis (MCA) of candidate SNPs segregates cow-collected mosquitoes from human-collected mosquitoes

**Fig. 4:**
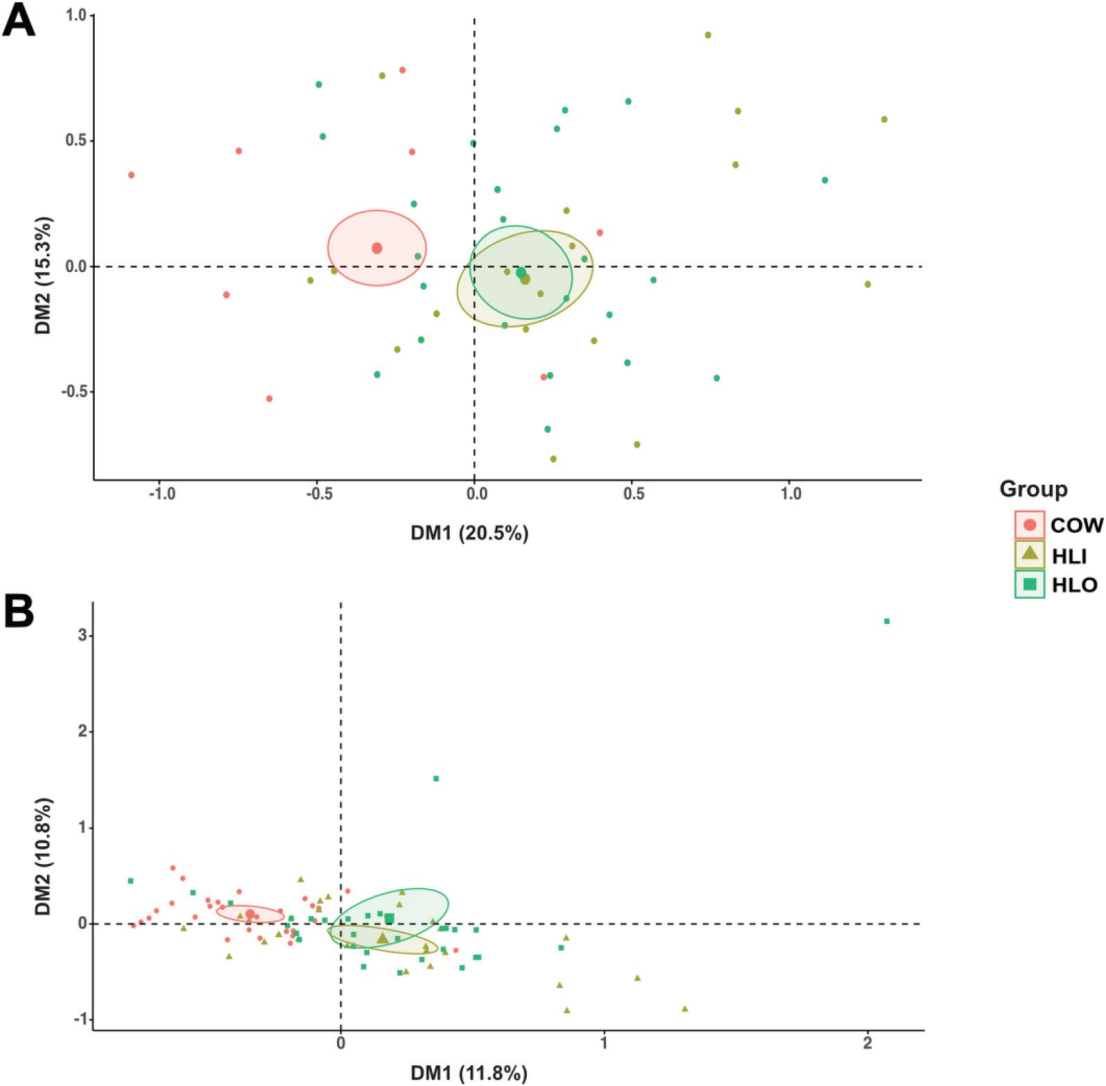
Multiple correspondence analysis (MCA) plot of candidate SNPs in 90 individual samples showing confidence ellipse around the mean point of variable categories. **A** Wild type versus mutated. **B** Wild type versus Homozygous SNP versus heterozygous SNP. *COW* cow-baited collection, *HLI* human landing indoor, *HLO* human landing outdoor.

**Fig. 5:**
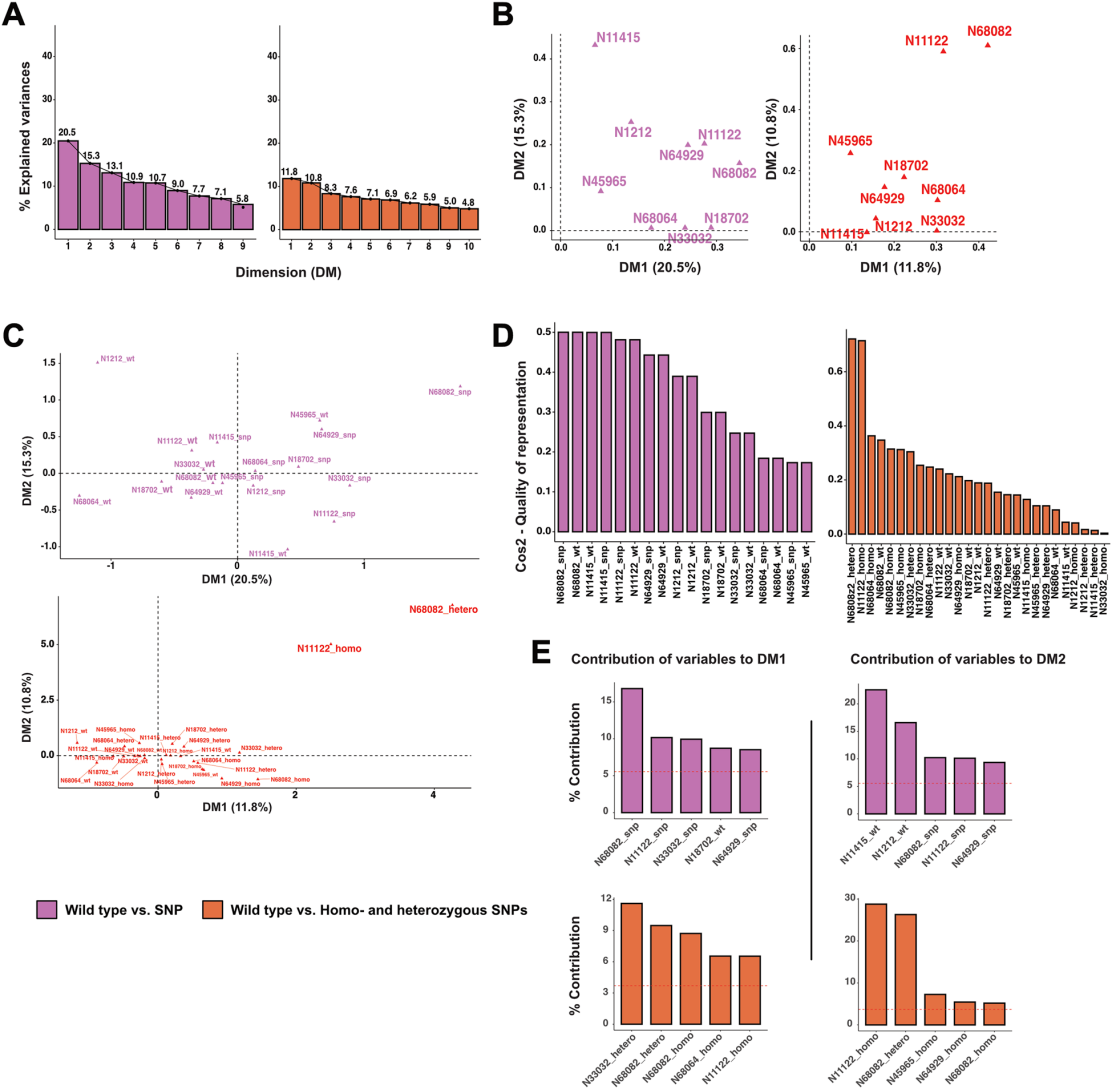
Multiple correspondence analysis (MCA) of candidate SNPs by wild type versus SNP (pink) and Wild type versus Homozygous SNP versus Heterozygous SNP (orange). **A** Eigenvalues of each dimension. **B** Correlation between candidate SNPs and principal dimensions. **C** Coordinates of candidate SNPs. **D** Bar charts showing quality of representation of variable categories using squared cosine (cos^2^) in descending order. **E** Bar charts showing top five contributions (%) of variable categories to dimension 1 (DM1) and dimension 2 (DM2).

To validate the whole-genome sequencing results, we designed specific primers targeting the identified nsSNPs and confirmed these variants by Sanger sequencing of the resulting amplicons (Tables S10 and S11). We then applied multiple correspondence analysis (MCA) to explore relationships among categorical SNPs and collection methods in the sequenced samples (Table 3). When coding each locus as wild type (WT) or SNP, individuals collected with human bait (HLI and HLO) clustered more closely together than those from the cow-baited group, as indicated by the confidence intervals (Fig. 4A). The first two MCA dimensions (DM1, dimension 1; DM2, dimension 2) explained 20.5% and 15.3% of the variance, respectively (Fig. 5A). In the correlation plot of variables versus dimensions (Fig. 5B), SNP68082, SNP11122, and SNP18702 showed the strongest association with DM1, while SNP11415, SNP1212, and SNP11122 were most closely linked to DM2. The variable-category coordinates (Fig. 5C**)** revealed that similar categories group together, with most WT points appearing in opposite quadrants, reflecting their negative correlations with the dimensions. We assessed the quality of representation for each variable category via the squared cosine (cos^2^) values (Fig. 5D); categories with a total cos^2^ near one are well represented by the first two dimensions. Notably, SNP68064 and SNP45965 had lower cos^2^ values, suggesting caution when interpreting their positions. To quantify each category’s contribution to the dimensions, we plotted percentage contributions in bar charts (Fig. 5E): SNP68082, SNP11122, and SNP33032 contributed most to DM1, whereas WT11415, WT1212, and SNP68082 were the top contributors to DM2. Finally, by replotting the MCA using three genotype categories (WT, homozygous SNP, heterozygous SNP; Fig. 4B), we observed a similar clustering pattern: HLI and HLO remained close, while the COW group formed a distinct cluster. In this plot, the dimensions accounted for 11.8% (DM1) and 10.8% (DM2) of the variance (Fig. 5A). Overall, these results support an association between selected olfactory gene SNPs and mosquito collection methods; however, this association requires further functional validation.

**Table 3:** Multiple correspondence analysis (MCA) results of variables and variable categories in the dimension 1 (DM1) and dimension 2 (DM2)

	WT versus SNP	WT versus Homo- and heterozygous SNPs
% Eigenvalues of DM1	20.5	11.8
% Eigenvalues of DM2	15.3	10.8
Correlation between variables and DM1	SNP68082, SNP11122, SNP18702	SNP68082, SNP11122, SNP33032
Correlation between variables and DM2	SNP11415, SNP1212, SNP11122	SNP68082, SNP11122, SNP45965
Most contributed categories of DM1	SNP68082, SNP11122, SNP33032	SNP33032_hetero, SNP68082_hetero, SNP68082_homo
Most contributed categories of DM2	WT11415, WT1212, SNP68082	SNP11122_homo, SNP68082_hetero, SNP45965_homo

## Discussion

This study represents the first investigation of genomic variation associated with biting behavior in *An*. *minimus* on the Thai–Myanmar border. Behaviorally, *An*. *minimus* showed the strongest anthropophilic tendency among local malaria vectors, as evidenced by its higher capture rate in human landing catches. It has been proposed to be the most anthropophilic *Anopheles* species in this area [58, 61, 85]. Although *An*. *minimus* populations in the study area shared similar ITS2 sequences, their *cox1* sequences were highly divergent, clustering into two sympatric lineages (A and B). This pattern likely reflects mitochondrial introgression within the complex, facilitating the transfer of novel alleles between taxa [86–90]. The low to moderate levels of genetic differentiation between lineages in this study are reminiscent of the past works in *An. arabiensis* [91], *An*. *maculatus* [92], *An*. *minimus* [66], *An. sinensis* [93], and *An. subpictus* [94]. They speculated the lack of geographical barriers resulting in gene flow between groups might describe this phenomenon. Further studies are needed to determine whether the two *cox1* lineages of *An*. *minimus* populations are conspecific or distinct biological species.

The lack of information on blood-meal origin may hinder our understanding of how specific olfactory receptor genes drive host-seeking behavior. Blood engorgement in mosquitoes induces dramatic physiological and behavioral changes, including selective downregulation of AgOr1 mRNA expression in *An*. *gambiae* at 12 h post-blood feeding [95] and altered chemosensory gene expression profiles in post-blood fed *Ae. aegypti* [96]. Determining the prior blood meals of field-collected specimens remains challenging when focusing on the recency of host-seeking behavior. Moreover, multiple blood feedings in a single gonotrophic cycle and high level of behavioral plasticity are common in *Anopheles* mosquitoes [97–100]. To control those factors, exploitation of an olfactometer to quantify host preference of *An*. *minimus* females from both field and lab strains will be essential for studying this complex behavior.

The idea that genes influence host-seeking behavior has arisen, and previous studies in other mosquitoes posits this hypothesis. In laboratory research, most findings point to the interruption of olfactory genes can alter the host preference in model mosquitoes under controlled conditions as previously mentioned. Field studies are more challenging due to numerous uncontrolled variables. Nonetheless, genetic differentiation associated with distinct feeding phenotypes has been documented in the *An*. *gambiae* complex, including chromosomal inversions linked to anthropophily [101–104]. Our findings demonstrate the differential nsSNPs between behavioral traits in the realm of host selection. Moreover, we identified over 2,000 significant nsSNPs in the sample genomes and the strong enrichment of olfactory-related gene families, calling into question the correlation between the presence of such SNPs and host-seeking behavior in this mosquito. Consistent with this hypothesis, our genome-wide results are relative to the findings of SNP markers in *An*. *darlingi* with different biting behavior (indoor versus outdoor and dusk versus dawn) [105]. The scaffold-level assembly of the *An*. *minimus* genome currently limits genome-wide analyses. A fully assembled chromosome-level reference would enhance future investigations.

We demonstrated that the allele frequencies of differential nsSNPs differ markedly among biting phenotypes. Because most selected SNPs and genes for validation were newly identified in this population, comprehensive gene annotation with orthologs was performed to characterize these genes and obtain detailed functional information. The three orthologous odorant receptors, Or1-like, Or65, and Or39, have all been experimentally shown to respond to specific ligands found in host-derived compounds. AgOr1 and AcolOR39 have been shown to be responsive to 4-methylphenol [106] and sulcatone [107], respectively. These compounds can be found in human body odor [108, 109]. The highly tuned receptor Or65 elicits a strong response to 2-ethylphenol, a component of animal urine [104]. Variation in olfactory gene families may underlie the evolution of host preference in insects [21, 110–114]. The *Or4* from field-collected *Ae. aegypti* is upregulated significantly in the human-biting colonies compared with the animal-preferring subspecies [52, 115]. This odorant receptor is also sensitive to sulcatone found in human odor. Another example of genetic changes involved in host preference is seen in the *An. farauti* complex, where amino acid changes in olfactory genes including *Ir8a* and *Or75* owing to selective pressure are associated with the shift from anthropophily to zoophily [116]. Many studies also suggest genes encoding odorant-binding proteins and antennal proteins, which are crucial in insect olfaction as the first recognition site and the carrier of odorant molecules [117–120]. Exploring the gene expression of candidate olfactory genes and other families along with SNP knockout or allele-specific editing will further elucidate the genetic mechanisms governing host-seeking behavior.

## Conclusions

Our findings provide the genetic and behavioral insights involved in host-seeking behavior in the primary vector of malaria, *An*. *minimus*, in Thailand. Polymorphisms in genome, especially olfactory genes, may contribute to altering mosquito feeding behavior. Nonetheless, impact of the surrounding environment has yet to be addressed. Further studies should be undertaken to determine the expression of olfactory genes in parallel with functional validation. This knowledge could form the foundation for the novel vector control management.

## Supplementary Information

Supplementary material 1. Table S1. *Anopheles* abundance collected from this study.

Supplementary material 2. Table S2. The unique haplotypes from *cox1* sequencing with accession numbers.

Supplementary material 3. Tables S3–S5. List of significant differential nsSNPs in each group comparison.

Supplementary material 4. Tables S6–S9. List of significantly enriched GO terms of mutual nsSNPs between COW versus HLI and COW versus HLO comparisons.

Supplementary material 5. Table S10. List of novel designed primer sequences used for SNP validation.

Supplementary material 6. Table S11. SNP validation data by Sanger sequencing.

## Data Availability

Supplementary files are attached to this paper. The raw whole-genome sequencing data are available in the NCBI Sequence Read Archive repository, BioProject ID PRJNA1187263. The unique haplotypes from *cox1* sequencing are deposited in GenBank (accession numbers PV174626–PV174768). SNP filtering and statistical analysis scripts are available on GitHub at https://github.com/kpusawang/minimus-wgs. Additional raw data, plots, and scripts are also freely available at https://github.com/kpusawang/minimus-wgs.

## Abbreviations

COW: Cow-baited collection
GO: Gene ontology
GR: Gustatory receptor
HLI: Human landing indoor
HLO: Human landing outdoor
IR: Ionotropic receptor
LTI: Light trap indoor
LTO: Light trap outdoor
MCA: Multiple correspondence analysis
nsSNP: Non-synonymous single-nucleotide polymorphism
OR: Odorant receptor
SNP: Single-nucleotide polymorphism
WGS: Whole-genome sequencing
